# The Role of Melatonin to Ameliorate Oxidative Stress in Sperm Cells

**DOI:** 10.3390/ijms242015056

**Published:** 2023-10-11

**Authors:** Achilleas Makris, Alexandra I. Alevra, Athanasios Exadactylos, Serafeim Papadopoulos

**Affiliations:** Hydrobiology-Ichthyology Laboratory, Department of Ichthyology and Aquatic Environment, University of Thessaly, Fytokou Str., 38446 Volos, Greece; acmakris@uth.gr (A.M.); aalevra@uth.gr (A.I.A.); exadact@uth.gr (A.E.)

**Keywords:** oxidative stress, melatonin, receptors, heat stress, artificial reproductive techniques, cryopreservation, pollution, sperm

## Abstract

It is widely accepted that oxidative stress (OS) coming from a wide variety of causes has detrimental effects on male fertility. Antioxidants could have a significant role in the treatment of male infertility, and the current systematic review on the role of melatonin to ameliorate OS clearly shows that improvement of semen parameters follows melatonin supplementation. Although melatonin has considerable promise, further studies are needed to clarify its ability to preserve or restore semen quality under stress conditions in varied species. The present review examines the actions of melatonin via receptor subtypes and its function in the context of OS across male vertebrates.

## 1. Introduction

The term stress means both systemic and local stress that is created after a disturbance of homeostasis. Usually, a specific stressor causes a specific type of local stress; meanwhile, when the intensity of stress exceeds a threshold, the hypothalamic–pituitary–adrenal axis is activated, resulting in the systematic stress response [1,2]. Additionally, as the concept of homeostasis becomes more specific, so does the concept of stress [3]. As an example, oxidative stress (OS) refers exclusively to the disruption of redox signaling and control [4,5].

Our common knowledge on oxidative stress has evolved substantially over the years, being focused mostly on the fundamental chemical reactions and the most relevant chemical species [6]. Thus, reactive oxygen species (ROS) and reactive nitrogen species (RNS) were identified as key players in initiating, mediating, and regulating the cellular and biochemical complexity of oxidative stress either as physiological or as pathogenic processes [6]. RS (reactive species) is defined as the disruption of redox signaling and control caused by RONS (both ROS and RNS), while the OS response is defined as the efforts made to restore homeostasis. This response ultimately leads to specific biological consequences that constitute the effects of this stress [3].

RONS are characterized as reactive molecules and can damage various biomolecules such as proteins, lipids, and nucleic acids [7]. The first studies conducted on the effect of RONS showed that they cause the pathogenesis of various diseases and the aging process [8]. Although excessive production of RONS can cause damage, when present in certain concentrations, they are essential for the regulation of life processes [7,9,10,11]. Consequently, many researchers conclude that an optimal level of stress is vital for maintaining health, as its absence can impair growth body composition, and development as well as lead to pathological conditions [1].

Male infertility has been linked to various types of stress as well as stressful life events [12,13,14,15]. Several studies, for example, have linked male (sub/in) fertility to stressful lifestyles [12,16,17,18]. Because mitochondria are the major energy source for sperm, they have been suggested as an important link between sperm motility and their response to stress [1,17,19,20]. Additionally, stress hormone receptors activate specific signaling pathways each time that are particularly important for both mitochondrial network homeostasis and sperm functionality [14,15,21]. Therefore, mitochondria are a particularly important organelle that contributes to sperm functionality and fertility [17].

Many strategies (using different sources of antioxidants) have been suggested to prevent the generation of oxidative agents. Among them, the use of melatonin (N-acetyl-5-methoxytryptamine), the principal hormone secreted by the pineal gland, has been suggested as a free radical scavenger and antioxidant [22]. Mainly due to its amphiphilic nature that allows it to pass through all morphophysiological barriers of the cell, it is one of the most effective antioxidants protecting cells from OS caused by reactive species [23]. In addition, its lipophilic nature allows it to easily cross cell membranes and act directly in various organs including those of the reproductive system [24,25]. Of particular interest is the fact that melatonin’s metabolites, which are formed when the hormone functions as a scavenger, are likewise equally as good or better than the parent molecule in neutralizing toxic oxygen-based and nitrogen-based reactants [26].

In this review, we describe the adverse influence of different stressful stimuli (e.g., heat stress, assisted reproductive technologies, pollution, and cryopreservation) on semen quality and its relation to mitochondrial function in the semen of various animals, from humans and fishes. Furthermore, the role of melatonin in ameliorating OS in sperm cells and future research directions are discussed.

## 2. Sources of RONS in Semen

There is a plethora of sources that produce reactive oxygen species (RONS) in semen. Among them are different qualities of sperm (immature, dead, and dysfunctional), organelles such as mitochondria, and factors associated with lifestyles and environmental agents.

Leukocytes: Leukocytes in small quantities are a normal finding in ejaculation and often pass through the male genital tract. The World Health Organization defines leukocytospermia as peroxidase-positive leukocyte concentration >1 × 10^6^ per mL of semen, and it occurs in about 10–20% of infertile men [27,28]. Polymorphonuclear neutrophils are the most common type of seminal leukocytes and the primary endogenous source of RONS in semen [29]. The activation of these leukocytes during genitourinary or accessory gland infections provokes an important increase in oxygen consumption and RONS generation [30,31].

Immature sperm: When there is a lot of cytoplasmic retention along the midpiece of the spermatozoa, there is a risk of functionally defective or immature sperm. These cytoplasmic residues contain an increased number of enzymes, particularly glucose-6-phosphate dehydrogenase (G6PDH) and NADPH oxidase [32]. The excessive presence of G6PD results in the generation of a significant amount of NADPH, which represents a primary source of electrons that are necessary for the reduction in molecular oxygen to O_2_•^−^ [31]. Several studies have suggested a correlation between cytoplasmic droplet retention and severe peroxidation damage to the sperm membrane. Creatinine kinase (CK) and G6PDH were used as biomarkers of this retention [33,34,35,36]. In addition, it has been reported that in an environment of increased RONS levels, immature sperm can cause OS in neighboring mature ones during their transit through the epididymis [37,38,39]. Also, mature sperm could be an additional source of RONS due to cellular metabolism [40].

Lifestyles and environmental factors: A list of factors associated with lifestyles and environmental agents could contribute to the severe generation of RONS in sperm. Cigarette smoking, alcohol consumption, electromagnetic radiation, obesity, microorganism mutations, or sexually transmitted diseases are actively involved [28,41,42,43].

Mitochondrial metabolism: Mitochondria are an important source of RONS, but at the same time, they have a system to control the levels of RONS to ensure that they do not exceed some critical limits and do not cause cell damage or apoptosis [44]. Superoxide dismutase is one of the most studied enzymes involved in mitochondrial RONS scavenging. Although excessive production of RONS is associated with OS, their presence at specific levels aids many cellular processes, while their lack in developmental and somatic cells can lead to impairments in cell differentiation, immune response, and autophagy [45,46,47].

Dysfunctional sperm: This kind of sperm presents excess residual cytoplasm, retention of histones, and poor protamination, resulting from differentiation failure during spermiogenesis [48]. There is a positive correlation between RONS production, anomalies in sperm morphology, and DNA damage [31,38,49]. High levels of expression of the isoform of NADPH oxidase (NOX)—NOX5, generators of RONS in sperm—have been reported in both teratozoospermia [50] and asthenozoospermic human ejaculates [37].

Dead sperm: Increased levels of dead spermatozoa in ejaculation are alarming during techniques of assisted reproductive technologies (ARTs) related to sperm, especially during cryopreservation. Dead sperm are high RONS producers [51], and their contribution to RONS generation varies according to the species. Dead sperm produce RONS through an aromatic L-amino acid oxidase (LAAO) pathway in bull semen [52]. In male species such as bulls, rams, and stallions, L-amino acid oxidase is increased following sperm death [29,52,53,54,55].

## 3. Effects of RONS on Sperm

Redox balance is substantial for maintaining different crucial factors of semen functionality [56]. On the other hand, an imbalance of redox status negatively affects semen quality due to oxidative damage [32]. Prolonged exposure and/or increased RONS concentrations cause extensive injury to miscellaneous biomolecules, such as proteins, lipids, and nucleic acids, which in the end hinder multiple cellular functions [30,32,40,57]. The adverse events include mitochondrial dysfunction, impaired sperm motility, loss of membrane integrity, DNA damage, and apoptosis [40,58]. A significant increase in RONS levels has been detected in 25–40% of semen samples of men who are infertile [59,60]. The severity of oxidative injury to sperm among infertile men depends on the length of exposure, concentrations, properties of reactive molecules, and antioxidant efficiency as well as surrounding temperature and oxygen tension [56]. In farm animals, the impact of OS is associated with reduced sperm quality and fertility [61,62]. Recently, Wyck et al. [63] found that bull sperm exposed to OS showed increased DNA damage, which was associated with impaired DNA demethylation during epigenetic reprogramming in early embryonic development. Ribas-Maynou et al. [64] found that the impact of OS at the sperm level translates into poorer fertilization and blastocyst rates after ARTs, such as in vitro fertilization (IVF) or intracytoplasmic sperm injection (ICSI).

The sperm plasma membrane consists of large amounts of polyunsaturated fatty acids (PUFAs), especially docosahexaenoic acids, which improve its fluidity that is necessary for multiple membrane fusion events [56]. At the same time, however, these fatty acids make sperm more susceptible to oxidative damage as they act as potential peroxidation substrates because they contain methylene groups with highly reactive hydrogen atoms [65]. There are marked differences among species in the composition of a sperm plasma membrane PUFAs. The most abundant PUFAs is either DHA (ω-3 PUFAs) or DPA (ω-6 PUFAs). From a broad perspective, differences in the sperm plasma membrane lipid composition among species explain differences in the response to semen processing (e.g., during cooling and cryopreservation) [66]. These differences may be attributable to variations in plasma membrane compositions [67], for instance, the content of lipids in the bilayer, degree of hydrocarbon chain saturation, cholesterol/phospholipids ratio, and protein/phospholipid ratio [68].

In addition, studies have shown that the excessive presence of RONS, especially nitric oxide (NO•), O_2_•^−^, and OH•, leads to the attachment of the above molecules to sperm DNA, causing excessive modifications and deletions in nucleotide bases and strand breakages, along with other multiple genotoxic effects. Sperm DNA, due to poor chromatin compaction and incomplete protamination, is particularly susceptible to oxidative damage [43,69]. Damage to sperm DNA due to RONS was experimentally demonstrated by the positive correlation between sperm ROS levels and sperm DNA fragmentation in humans [70,71].

RONS have also been considered one of the main apoptotic stimuli triggering mitochondria to produce specific signaling molecules crucial for the activation of programmed cell death [72]. More specifically, mature sperm from patients with elevated RONS levels showed intense apoptotic activity compared to the control group [73]. Reduction in this activity due to OS can be observed after antioxidant therapy with cinnamon bark oil consumption in asthenozoospermic men [74].

Several studies have correlated OS markers with fertility [75,76,77] and important sperm quality parameters such as morphology [78,79] and motility [80,81] in humans. Rapid progressive sperm motility is a highly sensitive indicator of lipid peroxidation and can be detected before the deterioration of other sperm kinematic parameters [56]. Additionally, the negative correlation between intracellular sperm O_2_•^−^ levels and average-path velocity (VAP) is important for understanding the potential role of free radicals in limiting the actual rate of sperm forward movement within the female reproductive tract, which is vital for successful fertilization [82]. Likewise, the positive correlation between superoxide dismutase (SOD) activity, curvilinear velocity (VCL), and amplitude of lateral head displacement (ALH) led to a quantification of the role of OS in the development of spontaneous and premature hyperactivated sperm motility during ejaculation [82]. Although a clear link between oxidative stress and male fertility disorders has been demonstrated in humans and laboratory animals, less information is available about the implications of this condition in the male fertility of domestic animals [83,84].

## 4. The Role of Melatonin

The major pathway of melatonin biosynthesis consists, in any organism or cell type tested, of tryptophan 5-hydroxylation, decarboxylation, *N*-acetylation, and *O*-methylation, in this order. Alternately, but at lower flux rates, melatonin can be formed via *O*-methylation of serotonin and subsequent *N*-acetylation of 5-methoxytryptamine or by *O*-methylation of tryptophan followed by decarboxylation and *N*-acetylation [85]. 

Melatonin has an impact working as either an antioxidant [86] or as a hormone due to its amphiphilic properties. Melatonin was discovered as a secretory product of the pineal gland and is now known to be generated in many organs in the body [87,88]. The hydrophilic and lipophilic properties of melatonin permit it to rapidly pass through all morphophysiological barriers easily and disperse in organs and fluids [89,90]. The important advantage of melatonin among other antioxidants is that in addition to melatonin itself, its metabolic derivatives formed during antioxidant reactions, including cyclic 3-hydroxymelatonin (3-OHM), N1-acetyl-N2-formyl-5-methoxykynuramine (AFMK), and N1-acetyl-5-methoxykynuramine (AMK), are all excellent free radical scavengers that contribute to the reduction in OS [26,88,91].

Melatonin involves multiple mechanisms to control cellular physiology, including via membrane receptors, nuclear binding sites, and interaction with cytosolic molecules [92]. Melatonin exerts most of its major physiological actions by reacting with the two membrane receptors MT1 and MT2, which belong to the G protein-coupled receptor superfamily [92,93,94]. 

The receptors of melatonin are expressed in several central nervous tissues and numerous peripheral tissues, including the testis and ovary [95]. Specifically, melatonin is involved in the modulation of the hypothalamic–pituitary–gonadal (HPG) axis, which is a very important regulatory center for animal reproduction, both in seasonal breeding animals and in non-seasonally breeding animals, including humans [96]. Some investigations have revealed that the MT1 receptor is widely distributed in endocrine tissues and brain regions, which are the main response sites of melatonin-induced physiological and circadian effects [97]. However, the MT2 receptor is generally absent in the mammalian hypothalamus and pituitary gland [97]. It is obvious that MT1 receptor is the more important receptor for melatonin-modulated reproductive regulation in mammals.

To date, the use of melatonin in human physiology has been restricted to the improvement of sleep quality, the alleviation of feelings of jet lag, and the reduction in sleep onset latency [98]. However, numerous studies have concluded that melatonin could also be associated with the prevention of several diseases related to aging and oxidative stress, including type 2 diabetes, cardiovascular and neurodegenerative diseases, or cancer [99,100,101,102,103,104,105,106]. Simple and composite formulations of synthetic melatonin come in a wide range of forms such as tablets, pills, sublingual drops, liquids, gels, creams, and even suppositories, as well as at dosages from 0.1 mg to 400 mg. In the case of composite formulations, synthetic melatonin is presented together with other compounds such as tryptophan, vitamins (C and B6), minerals, and even collagen and hyaluronic acid in the case of creams [98].

### 4.1. Melatonin Receptors

In vertebrates, there are two melatonin receptοrs: MT1 (or *Mel1a*, gene *MTNR1A*) and MT2 (or *Mel1b*, gene *MTNR1B*) [107]. The melatonin receptor family belongs to the class A rhodopsin-like G protein-coupled receptor (GPCR) subfamily [94,95]. The MT1 receptor is composed of 350 amino acids, while the MT2 receptor is composed of 362 amino acids, showing 55% and 70% homology in the transmembrane domain [108]. Interestingly, ruminants such as cows, sheep, and camels have MT1 and MT2 receptors that are about 10 amino acids longer than those in humans [109], although there is no report of significant functional differences among species. The two receptors have 60% homology among mammalian species, including humans, rats, mice, and ruminants [110,111]. An additional member, MT3, also termed *Mel1c*, has been identified but only in nonmammalian species, such as birds, amphibians, and fish [96,112]. 

### 4.2. Melatonin Subtype Receptors

Melatonin’s effects on reproduction are likely acting as a hormone that binds subtype receptors [86]. Differences in regulatory functions of melatonin-binding proteins have procured categories of melatonin receptor subtypes [113]. Melatonin subtype receptors include membrane G protein-coupled receptors and nuclear orphan receptors [113]. There is scientific evidence that nuclear orphan receptors might be primary targets downstream in the membrane receptor signaling pathway [114]. Also, many studies have proven that melatonin’s functions via membrane receptors regulate metabolic, cardiovascular, immune, and reproductive systems [115].

### 4.3. Melatonin-Receptor-Mediated Biological Effects

**Figure 1:**
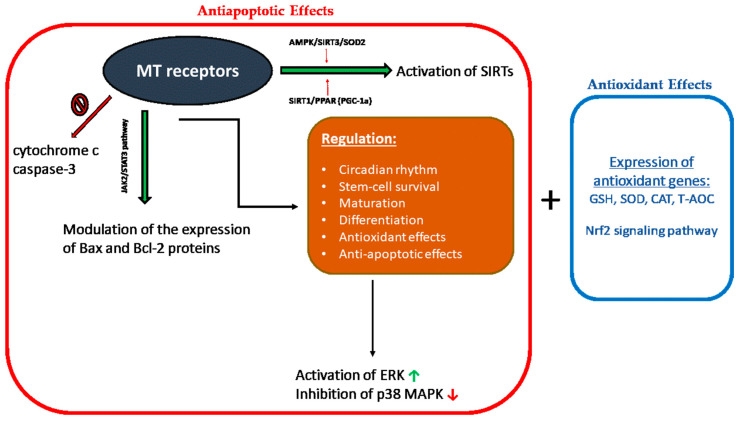
Illustration of the biological effects that are mediated with melatonin receptors. Red frame (antiapoptotic effects) and orange frame (antioxidant effects). MT: melatonin; SIRTs: certain histone deacetylases; Nrf-2: nuclear factor erythroid 2-related factor; GSH: glutathione peroxidase; SOD: superoxide dismutase; CAT: catalase; T-AOC: total antioxidant capacity; ERK: extracellular signal-regulated kinase; and p38 MAPK: mitogen-activated protein kinases.

Melatonin receptors play a significant role in miscellaneous physiological activities such as the regulation of circadian rhythm, stem-cell survival, maturation, differentiation, antioxidation, apoptosis, and cancer [116]. Melatonin receptors are also involved in antioxidant and antiapoptotic effects [117]. The biological effects that are mediated with melatonin receptors are represented schematically in Figure 1.

#### 4.3.1. Antioxidant Effects

The effects of melatonin and its metabolites on RONS levels and mitochondrial protection are expected to be linked to the activation of basic signaling pathways such as stress-activated/mitogen-activated protein kinases (SAP/MAPKs). These protein kinases are mediators of signal transmission from the cell surface to the nucleus and practically control most of the physiological activities in eukaryotic cells [118]. Nuclear factor erythroid 2-related factor 2 (Nrf-2) and heme oxygenase-1 1 (HO-1) regulate the antioxidant potential of cells by regulating the transcription and translation of various antioxidant enzymes [119].

Melatonin protects the cell from OS by inducing the expression of antioxidant genes such as glutathione peroxidase (*GSH-Px*), superoxide dismutase (*SOD*), catalase (*CAT*) and by increasing the total antioxidant capacity (T-AOC) in the presence of a stimulation of respiratory complexes activity [120]. These effects are mediated by the stimulation of transcriptional proteins, such as nuclear factor erythroid 2 (Nrf2), the activity of which is particularly important for the cryoprotection and stress adaptation effects of melatonin [121].

#### 4.3.2. Antiapoptotic Effects

Melatonin modulates the expression of the proteins Bax and Bcl-2 and inhibits the release of cytochrome c and the activation of caspase-3. Melatonin-induced Bax/Bcl-2 translocation occurs via the JAK2/STAT3 pathway, and its antiapoptotic effect is mediated via ERK activation and p38 MAPK inhibition [122,123,124]. In addition, melatonin-activated signaling cascades also include activation of certain histone deacetylases (SIRTs) through AMPK/SIRT3/SOD2 and SIRT1/PPAR- coactivator (PGC-1α) [125,126]. The regulation of transcription factor PGC-1α is MT1-receptor-dependent [127]. The blockage of melatonin receptors significantly promotes hCG-induced apoptosis in mice [109]. 

### 4.4. Melatonin Effects on Testicular Physiology

Sperm motility in humans seems to be unaffected by melatonin [128], inhibited by high concentrations of melatonin in Wistar rats [129]. These varying effects of melatonin on sperm motility might be due to the time of day the tissue was harvested, photoperiodicity of these mammals, or even melatonin-receptor expression in sperm [113]. Melatonin implants in Rasa Aragonesa rams during the non-reproductive season (spring) increased seminal plasma testosterone (T) concentrations after four weeks and 17α-estradiol (E_2_) after eight weeks [130]. From this, we can predict that for seasonally breeding males that respond to melatonin to time their breeding, melatonin administration can override other endogenous circannual rhythms to activate the mammalian reproductive axis [113]. Melatonin administration for fifteen weeks during the breeding season compared to the non-breeding season significantly increased plasma T concentrations in both winter and spring seasons in Chios rams, and this increase was higher in winter [131]. In Syrian hamsters (long-day breeders), testes collected at different reproductive states (photostimulated and photorefractory) showed that melatonin concentration is significantly higher in the testes of hamsters kept on short days (photorefractory) relative to their long-day (photostimulated) counterparts [132].

In addition to the implication of seasonal changes in the synthesis of melatonin in testes, Mukherjee and Haldar [132] also found that the MT1 receptor was significantly higher in testes collected from the short-day group. Across a variety of breeding types as long-day, short-day, and non-seasonal breeders [133], MT1 and MT2 were expressed in the sperm of these types in a receptor-mediated manner affecting semen [113]. In Table 1, the effects of melatonin supplementation in different media for different species on sperm cells are summarized.

### 4.5. Melatonin and Sperm Quality

Melatonin is now considered a potential improver of sperm motility [107]. In in vivo experiments performed on mammals such as rams [140], bucks [141], and buffalos [142], subcutaneous implantation of melatonin resulted in better sperm quality and improved both total and rapid motility as well as sperm viability. In vitro experiments with melatonin showed a significant reduction in sperm deformations and an improvement in its stability, viability, and fertilizing ability, including non-sorted and sex-sorted sperm [143,144].

Despite the positive effects of melatonin due to its antioxidant properties and its receptor-mediated signaling transduction, its mechanism of action is still not fully clear [107]. This hormone can scavenge high levels of RONS in sperm, thereby reducing DNA damage and sperm apoptosis. It can also remove nitrogen-based reactants and toxic oxygen but also increase the activities of antioxidant enzymes, such as catalase (CAT), glutathione peroxidase (GPx), and superoxide dismutase (SOD) [107]. 

**Figure 2:**
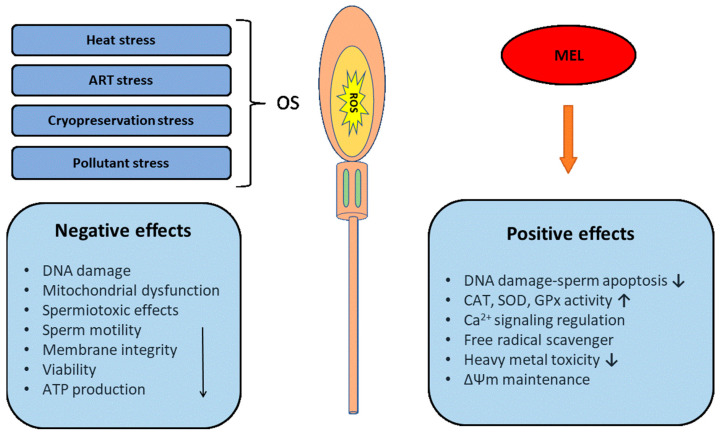
Illustration of the negative effects of different types of stress and the positive effects of melatonin (MEL) on sperm. ROS: reactive oxygen species; SOD: superoxide dismutase; CAT: catalase; GPX: glutathioneperoxidase; and ΔΨm: mitochondrial membrane potential.

Melatonin has improved the mobility and velocity parameters of sperm because of its interaction with calmodulin [145], which influences the cytoskeletal elements of the sperm. Melatonin also stimulates the cellular influx of Ca^2+^ into sperm cells enhancing their motility [146]. Calcium and its channels control the transition of flagella wave forms from symmetric to asymmetric [147]. Calmodulin acts as an intracellular regulator of Ca^2+^ function and has been identified both in the head and flagellar parts of spermatozoa [148], and it is believed to be a major signal transducer of Ca^2+^ in regulating motility. Additionally, it modulates the levels of the second messenger cAMP [149], which acts both via the axoneme of the sperm tail [150] and via cell-membrane-dependent pathways [151], thereby improving sperm motility and velocity. The negative effects of different types of stress and the positive effects of melatonin (MEL) on sperm are illustrated in Figure 2.

## 5. Melatonin Alleviates Heat-Stress-Induced OS

### 5.1. The Problem of Heat Stress Conditions

In the case of heat stress (HS), an increase in RONS levels is caused due to the initiation of OS [152]. Sperm functionality is negatively affected by OS due to the reduction in motility parameters, DNA integrity, and mitochondrial dysfunction [152]. Characteristically, HS induces dysfunctions in mitochondria involving reduced activities of the mitochondrial respiratory chain (complex I and IV) and sperm membrane potential, leading to reduced ATP content [152].

Gong et al. [153] reported that under HS conditions, sperm mitochondrial function is affected mainly due to interferences in mitochondrial remodeling and mitochondrial protein transport. Additionally, HS affects the induction of glycogen synthase kinase 3 (GSK3) activity, which modulates the permeability of the exterior membrane of mitochondria, leading to decreases in the mitochondrial ability to release ATP [153]. Furthermore, increased mitochondrial ROS production has been linked to decreased sperm motility and loss of mitochondrial membrane potential (MMP) [154]. Specifically, Rahman et al. [155] induced heat stress in cryopreserved bovine semen by exposing it to 41.0 °C and observed a decrease in plasma membrane integrity and mitochondrial potential. Finally, the abnormal function of sperm mitochondria due to heat stress reduces its functionality, leading to male infertility [156].

### 5.2. Melatonin’s Anti-Heat Stress Action

To enhance and improve sperm mitochondrial function due to adverse environmental stress such as HS, several antioxidants have been proposed and used [77,139,157,158] In addition, an extensive study of the negative effects of HS on sperm mitochondrial function could help develop new techniques to mitigate them using mitochondrial enhancers [157].

Zhao et al. [134] reported that 1000 μM of melatonin acts against HS-induced (the sperm aliquot was incubated in an incubator at 42 °C for 3 h in a humidified air atmosphere with 5% of CO_2_) oxidative damage in human sperm and specifically improves sperm motility, reduces mitochondrial RONS production, stabilizes mitochondrial membrane potential balance, reduces lipid peroxidation, helps maintain DNA integrity, and reduces apoptosis. Also, exposure of human sperm suspensions to 1000 µM of melatonin for 30 min led to an improvement in motility and a decrease in the number of immobile spermatozoa [135]. Accordingly, treatment with 2 mM of melatonin for 120 min in human sperm improved motility and reduced NO and ROS generation [136].

Recently, Qin et al. [137] reported that melatonin can effectively confront different endoplasmic reticulum stress (ERS) signaling pathways and reduce mouse spermatocyte apoptosis [159]. The ERS is a critically crucial factor in many pathologies, and several studies have linked ERS to heat stress [137]. Melatonin actions on the mouse spermatocytes during high temperature include the inhibition of signaling pathways *Atf6* and *Perk*, down-regulation of the expression of *Chop* and *Caspase12,* and reduction in apoptosis [137]. The role of melatonin in the mouse spermatocyte (cell line GC2) was dependent on both MT1 and MT2 receptors [137]. In contrast, melatonin activity in mammals was mediated through its interactions with the G protein-coupled MT1 and MT2 receptors [115].

Guo et al. [138] confirmed experimentally in vivo that exposure to HS causes loss of mouse germ cells (CD-1) and disruption of testicular histomorphology. Apoptotic germ cells were experimentally cleared, resulting in a reduction in all stages of these cells in the seminiferous tubules, leading to hollow seminiferous tubules and decreased testicular weight. Subsequently, the seminiferous tubules gradually filled with regenerated germ cells, and testicular weight was restored. The use of melatonin in the above experiment failed to prevent testicular damage due to HS but significantly accelerated testicular recovery [138]. The above work is the first report that showed that the prolonged use of melatonin accelerates the repair of heat-stress-induced testicular damage in mice. This action of melatonin promotes the clearance of apoptotic germ cells through RAC1-mediated phagocytosis via Sertoli cells and supports the regeneration of germ cells by restoring tight and gap junctions. Additionally, melatonin improves the phagocytotic capacity of Sertoli cells by upregulating the Cx43 gap junction protein Connexin43 [138].

In 2022, Shahat et al. [139] induced mild HS (scrotal neck isolation) in rams and studied whether melatonin use affected testicular blood flow and sperm quality. It was experimentally shown that from 2 to 7 weeks after stress induction, the group receiving melatonin (subcutaneously) showed higher and better progressive and total motility. Also, compared to the control group, it showed fewer sperm abnormalities and higher acrosome integrity. The above work is the first report of the positive effects of melatonin in testicular protection against HS in farm animals and confirms the improving role of this hormone in testicular blood flow as well as the protective role in sperm motility and morphology in rams that have been exposed to HS. There are also studies showing that injectable melatonin in rams [160] and goats [161] improved sperm quality during the non-breeding period. The above positive effects are considered to be due to the systemic or local action roles of melatonin in the testis but also to the increase in the blood flow of the testicles, which probably also acts as an initiation of their endocrine function [162]. Another theory claims that its improving effect is due to its antioxidant and antiapoptotic properties in the testicles and sperm [163]. Therefore, based on the above results, melatonin is considered a substance that can significantly mitigate the negative effects of testicular HS under field conditions. In Table 1, the effects of melatonin supplementation on sperm are summarized against the negative impacts of heat stress.

## 6. Assisted Reproductive Techniques (ARTs) and OS

### 6.1. Reproductive Technologies and OS

Sperm during ARTs receive a variety of in vitro manipulations in human and domestic animals [164]. Also, OS during ARTs could arise from several exogenous factors such as exposure to cryopreservation, visible light, O_2_ tension, centrifugation, culture media, pH, and temperature, which generates iatrogenic damage [164,165].

The results that the various reproductive techniques will give are affected by several factors, the most important of which is sperm OS. Potential oxidative damage to sperm DNA can lead to negative effects on embryo development or cause mutations in the offspring [166]. During ARTs, sperm are subjected to altered microenvironments, shearing forces, and a variety of exogenous factors that differ significantly from the physicochemical microenvironments of the female reproductive tract [164]. Sperm cryopreservation, laboratory handling, and washing techniques could increase the level of OS in sperm, especially in the case that the initial sperm ROS levels are already elevated [164].

Many studies have proven that the prolonged incubation of sperm during IVF (in vitro fertilization) procedures induces sperm DNA fragmentation (SDF) and OS both in human and animal models [167,168,169,170,171]. Characteristically, after incubation of sperm from healthy donors, reduced motility and increased RONS levels were observed starting from 6 h and reached a peak at 48 and 24 h, respectively [172].

Sperm concentration during ARTs also affects RONS levels, viability, and SDF in many species [164]. Post-thaw incubation of ram sperm at 6 to 100 × 10^6^/mL for 6 h increased SDF, with lower sperm concentrations responding better [173]. In bovine, the level of sperm concentration during the storage of semen in a fresh extender affects the level of RONS, with higher concentrations of sperm pressing the sperm cell viability and increasing OS compared to lower sperm concentrations [174].

Oxygen tension during an IVF procedure could influence the levels of ROS in sperm. According to the usual method, sperm are incubated in 20% O_2_, but samples with high ROS levels could benefit from reduced O_2_ tensions [164]. A study reported no differences in sperm function for normozoospermic sperm samples after incubation under 5 versus 20% O_2_, whereas 5% O_2_ incubation was found beneficial for oligozoospermic samples that are known to have higher levels of ROS than fertile samples [175].

### 6.2. Beneficial Role of Melatonin

As an antioxidant, the use of melatonin, as a radical scavenger and antiapoptotic factor [176,177,178,179], has already been investigated. Indeed, many studies reported that melatonin reduced the rate of OS in human sperm, having beneficial effects on quality parameters, such as motility, DNA integrity, and lipid peroxidation [134,135,180,181,182,183,184].

Monllor et al. [184] suggested that certain levels of melatonin in the sperm environment from spermatogenesis to capacitation could be an effective strategy to avoid RONS-induced infertility. Melatonin, unlike other antioxidant compounds, reduces RONS levels to some extent, and low levels of RONS act as second messengers of capacitation [185].

Very recently, the use of melatonin to preserve human sperm quality, using cadmium (Cd) as an inductor of OS in vitro, was examined by Minucci and Venditti [186]. It is well known that testis is one of the main targets of this heavy metal, and many papers focused on its ability to generate OS, as well as other damages, compromising the normal spermatogenesis and decreasing sperm quality in both animal models [187,188] and humans [189,190]. Minucci and Venditti [186] further expanded the information concerning the protective action of melatonin when RONS generation is favored, using Cd as a pro-oxidative agent in human sperm in vitro. 

According to the above researchers, the negative effects of cadmium-induced OS were counteracted by co-treatment with melatonin. In particular, its use improved sperm motility after 30 min of incubation compared to the control group, and at 24 h, it prevented the physiological alteration in terms of motility, DNA integrity, and apoptosis [186]. The above data lead to the conclusion that melatonin can be used to improve the quality of human gametes under stressful conditions [186].

Caspase-3 participates in cell apoptosis, being a good marker of cell death, and active caspase-3 is exclusively found in the midpiece of spermatozoa [191]. According to Monllor et al. [184], the use of melatonin in the sperm incubation medium before ART (storage at 37 °C for 30 min), in both normal and oligozoospermic samples, prevented an increase in the activity of caspase-3 as well as subsequent DNA fragmentation. Although the mechanism of action of melatonin for the above positive effects is not yet known, Espino et al. [192] reported that melatonin could reverse the apoptotic events caused by OS or elevated intracellular Ca^2+^ levels.

In human sperm, the expression of CD46, which is located in the inner acrosomal membrane [193], has an important role in the fusion process of the spermatozoon with the oocyte membrane [194] and therefore is considered an indicator of the fertilizing capacity of the sperm [184]. Based on the above, Monllor and colleagues [184] studied the effect melatonin had on CD46 expression in sperm samples. The results saw that melatonin increased the ratio of adequate sperm both in normal and oligozoospermic groups.

During the late stages of spermatogenesis in mammals, the sperm nucleus acquires high levels of chromatin condensation [184] to protect DNA from oxidative damage. When sperm DNA exhibits reduced condensation, chromatin is much more susceptible to OS and deletions, mutations, DNA cross-links, and chromosomal rearrangements [195], and it has been associated with subfertility in several species [196]. Melatonin, although unable to improve DNA compaction during the short time of sperm preparation for ART, can enhance the migration of sperm with compacted DNA and prevent its fragmentation [184]. In Table 2, the effects of melatonin supplementation on sperm are summarized against the negative impacts of ARTs.

## 7. Effects of Melatonin on Cryopreservation-Induced OS 

### 7.1. Cryopreservation and OS

The cryopreservation of reproductive cells is known to be an important tool in human- and animal-assisted reproduction. Despite the benefits of cryopreservation, during this process, an imbalance is observed between the production of RONS and the antioxidant defense mechanism of the cells, known as OS [197]. Although all cells possess specific defense mechanisms against RONS, when these are overwhelmed, RONS can affect various cellular functions and processes [198]. Specifically, sperm are unable to cope with extreme RONS production mainly due to two reasons. The first is the low amount of antioxidant compounds due to the reduction in the cytoplasm in the final phases of spermatogenesis [199,200,201], and the second is the reduction in the antioxidant capacity of sperm due to the dilution of the seminal plasma, which is endowed with enzymatic and non-enzymatic antioxidants [202]. The above two reasons, as well as the high content of the sperm cell membrane in unsaturated fatty acids, make these cells vulnerable to RONS, leading to irreversible cell changes [203].

Oxidative stress is the main cause of most of the structural and molecular damages that occur in sperm during both freezing and thawing. Specifically, they lead to lipid peroxidation, DNA fragmentation, mitochondrial damage and dysfunction, protein oxidation, and loss or inactivation of enzymes associated with sperm motility [203]. OS is, also, one of the main factors associated with male infertility and reduced sperm viability and reproductive capacity. Additionally, OS has been linked to reduced rates of fertilization and in vitro embryo development in non-human mammals [29].

During the freezing–thawing process, motility is most affected [204,205], and this is due to mitochondrial damage and physical changes that occur in the tail of the spermatozoa [206]. In particular, the damage observed in the mitochondrial membrane interrupts the energy production process, thus reducing ATP production while the irreversible coiling of the flagellum caused by cryopreservation prevents the propulsive movement of the tail [206]. In addition, the greatest percentage of peroxidation and membrane damage takes place in the mitochondria, resulting in a reduction in ATP, loss of sperm motility, reduction in sperm–egg fusion, and DNA damage [202].

During the cryopreservation process, changes occur in the fluidity of the mitochondrial membrane, resulting in an increase in mitochondrial membrane potential (*ΔΨm*) and, by extension, the release of RONS [29]. In turn, these cause DNA damage leading to single/double-strand DNA breaks [29]. From ovine studies, it is implied that freezing–thawing injury to sperm mitochondria significantly reduces the ability of sperm to migrate through the cervix and survive in the female reproductive tract [29]. Accordingly, studies in bulls confirmed that mitochondrial dysfunction is due to the opening of the mitochondrial permeability transition pore in response to intracellular Ca^2+^ increases, and this was associated with the loss of *∆Ψm*, decreased ATP content, increased RONS levels, and deterioration of plasma membrane integrity [29].

According to Figueroa et al. [207], significant structural alterations were detected in the midpiece and mitochondria in the cryopreserved sperm of fish. These alterations reduce the functionality of the mitochondria and the energy reserves of the cell, creating problems in cellular osmoregulation, ion exchange, lipid peroxidation, and enzymatic mechanisms that regulate motility [207]. According to Cabrita et al. [208] and Figueroa et al. [209], the mitochondria of cryopreserved sperm in the species *Dicentrarchus labrax*, *Acipenser ruthenus*, *Cyprinus carpio* L., *Oncorhynchus mykiss*, *Salvelinus fontinalis*, and *Sparus aurata* show increased sensitivity to frost damage. Additionally, in trout and Atlantic salmon, mitochondria show 40–50% of the mitochondrial membrane potential, while Atlantic salmon shows 61% of the mitochondrial membrane potential [207]. A positive correlation between mitochondrial membrane potential and fertilization rate in *Onchorynchus mykiss* and *Salmo salar* has also been reported, a fact possibly related to the reduced motility and fertilization capacity presented by the cryopreserved sperm of these two species [207].

### 7.2. Beneficial Role of Melatonin

Melatonin has been shown to protect sperm from oxidative damage, maintain sperm viability, reduce morphological abnormalities, and prevent DNA fragmentation [29]. The in vitro use of this hormone can improve the quality characteristics of human, ram, and pig sperm, while its use as an antioxidant agent in cryopreservation improves the quality of sperm after thawing [197].

Alevra et al. [197] reported the effects of the hormone melatonin both on the cryopreserved sperm of humans and of productive animals and fish. The addition of melatonin to cryopreserved solutions of buffalos [210,211], bovine [212], sheep [213], human [214,215], fish [216,217,218,219], and pig [220] sperm increased its viability after thawing and reduced morphological abnormalities. Unlike sperm enrichment with melatonin, no differences were observed during cryopreservation in sperm quality of goat semen [221]. Specifically, in several studies, it was shown that the administration of melatonin on both fresh and frozen sperm improved membrane integrity, motility and velocity, capacitation, antioxidant protein quantity, and developmental competence of sperm [222,223,224,225,226,227,228,229,230,231,232,233,234].

In cryopreserved bull sperm, the administration of 0.25 mM of melatonin increased VAP and VSL velocities, while the administration of 0.1 mM protected the plasma membrane and acrosome region and maintained the ultrastructure integrity of the sperm [229]. The administration of 0.2 mg/mL of melatonin to Mediterranean buffalo sperm improved its antioxidant capacity, motility, and morphology during cryopreservation [230]. Furthermore, the addition of 1 mM of melatonin in swamp buffalo bull sperm protected it from damage during cryopreservation [231]. In frozen–thawed pig sperm, 1.0 μM of melatonin presented higher viability and acrosome integrity, lower levels of peroxynitrite, ⋅O^2−^, and lipid peroxidation and diminished the levels of total ROS [220]. On the other hand, melatonin supplementation in canine sperm had no effects on it [232].

Our group [219], tested the effects of melatonin (0.5 mM, 1 mM, 1.5 mM, and 2 mM) on sea bream (*Sparus aurata*) sperm, stored at −196 °C and 4 °C. During short-term storage (4 °C), the melatonin improved sperm motility, allowing the sperm to remain motile for a longer storage period compared to fresh sperm and the control group. On the contrary, in the cryopreserved semen, no improvement was observed in the kinematic parameters of sperm [219].

Melatonin has the ability to maintain *ΔΨm* and preserve various mitochondrial functions. This is achieved by scavenging ROS and RNS and inhibiting the mitochondrial permeability transition pore (mPTP) opening. In addition, this hormone has the ability to regulate the expression of various antioxidant enzymes and genes that deal with stress [29].

Studies have shown that the use of melatonin as an antioxidant agent in cryopreserved ram sperm inhibits mPTP opening, thus improving sperm motility and viability, ATP synthesis, and oxygen consumption as well as the function of key OXPHOS enzymes [233,234]. Moreover, in ram sperm, the use of melatonin led to the suppression of mPTP opening, thus protecting the mitochondria and improving its quality characteristics during cryopreservation [234]. Additionally, the use of this hormone during the sperm equilibration period before cryopreservation led to an increase in plasma membrane integrity, ΔΨ*m*, and mitochondrial Cyt C concentration, while correspondingly it helped to inhibit the mPTP opening and reduce enzymatic activity of Cyclophilin D (key mediator of the mPTP opening) [29]. Finally, the use of melatonin is shown to enhance the expression of the antiapoptotic genes Bcl-2 and heat shock protein 90 (HSP90), thereby conferring resistance to stressors in cryopreserved sperm [29]. In Table 3, the effects of melatonin supplementation on sperm are summarized against the negative impacts of cryopreservation.

## 8. Antioxidant Role of Melatonin against Environmental Pollutants

Reproductive functions and embryo development procedures appear to be affected by chemical compounds, which are known as endocrine disruptors, resulting in degradations during the reproductive process in various species of birds, reptiles, fish, and mammals [237].

Chemical elements, such as organotin, whose use is mainly for biocides in antifouling paints, are considered responsible for reproductive disorders in the male genital organs of marine gastropods [237]. According to an experiment that was carried out on two different marine invertebrates, some nanoparticles such as nickel and copper oxide had spermiotoxic effects [237]. More specifically, these nanoparticles had negative effects on several semen parameters, such as concentration, morphology, motility, mitochondrial potential, and the generation of OS, which in turn affected the membrane and genome integrity [237].

Ribeiro et al. [238] supported that several heavy metals have a toxic effect on the male reproductive system, causing a reduction in semen quality and the production of RONS. Metals, such as iron (*Fe*), Cadmium (*Cd*), and Mercury (*Hg*), have been associated with the reduction in progressive motility and other motility parameters in species such as buffalo, bull, ram, and goat [238].

Generally, in the male reproductive system, an essential role of melatonin is the prevention of testicular damage, and it is effective in maintaining the blood–testis barrier in testes [239]. Concerning heavy metals, it is likely that melatonin plays an essential role in the reduction of negative impacts caused by Cadmium (*Cd*) in the male reproductive system, improving the DNA damage and suspending autophagy in sperm through the ATM/AMPK/mTOR signaling pathway [240].

Based on several studies carried out in recent years on the use of melatonin against metal-induced cytotoxicity, it appears that melatonin has a protective role against OS by counteracting free radicals and enhancing the antioxidant enzyme activity [239]. 

According to Li and colleagues [239], melatonin can be effective in toxicity reduction, induced by metals such as copper, iron, and molybdenum, due to its high lipophilicity, low toxicity, and ability to penetrate fast into cells [239]. The same research group studied the exposure of haexavalent chromium in spermatogonia and the role of melatonin in the protection against haexavalent chromium toxicity. They prove that melatonin can eliminate RONS by suppressing ATM p53 phosphorylation and the mitogen-activated protein kinase (MAPK) [239].

Dehdari Ebrahimi et al. [241] studied 38 publications, 31 of which were included in meta-analysis. The results of most studies presented useful data on the role of melatonin in the histopathological features of testicular tissue. Specifically, 20 toxic compounds were studied, some of which belonged to heavy metals while others to environmental pollutants [241]. The general conclusion reached by the researchers of this review, studying the effects of various chemical compounds in animal models, is that melatonin treatment significantly improved spermatological parameters (increased sperm count, motility, viability, and spermatozoa diameter), serum steroid hormone levels (testosterone and luteinizing hormone), antioxidant enzyme levels (superoxide dismutase and glutathione), and sperm morphometrics (epididymis weight and testicular weight) [241]. Despite this, the action of melatonin was found to be at lower levels, in relation to the apoptotic index and the treatment of different morphological changes in the structure of the sperm [241]. 

Except heavy metals, melatonin (MEL) showed a strong antioxidant action against Bisphenol S (BPS), an endocrine disrupting chemical, which contributes to cell apoptosis and causes negative effects in testis histomorphology (histological degenerative changes) and sperm quality parameters in adult male golden hamsters (*Mesocricetus auratus*) [242]. Reduction in serum levels of testosterone, melatonin, and testicular oxidative stress (TOS) was also associated with ΒPS exposure [242]. During treatment with MEL (10 mg/kg BW/day), there was an improvement in sperm quality parameters (viability and sperm count), restoration of the testicles to their original state, enhanced activity of antioxidant enzymes SOD and CAT, and a decrease in proteins NF-kB/COX-2, which are responsible for inflammation [242]. Finally, MEL seems to act as a stimulator in signaling pathways Nrf-2/HO-1 and SIRT-1/ FOXO-1, which are responsible for regulating antioxidant proteins [242]. 

Regarding the effect of melatonin in minimizing the toxicity of heavy metals in the testicles, Venditti et al. [243] concluded for the first time that the co-administration of melatonin (3 mg MEL/L) to cadmium-exposed rats weakened cadmium toxicity against genes *DAAM1* and *PREP* expression in the testes [243]. In addition to this, the protective effect of melatonin against cadmium toxicity is confirmed by the reduction in oxidative stress and apoptosis as well as the restoration of histological and biomolecular changes (alterations in CAT and SOD activity) in normal levels, before causing damage to the testicles, due to exposure to cadmium [243]. It is considered appropriate to mention that the treatment exclusively used melatonin, and it was observed that this antioxidant agent is characterized by the ability to cause increased expression of *PREP*, which regulates the concentration of the hormone’s progesterone and GnRH, while its role is linked to microtubule processes [243]. Finally, the researchers presented new evidence on the mode of action of melatonin as a protective factor in other studied testicular parameters, such as in the improvement of impaired steroidogenesis (reduced protein level of StAR and 3β HSD) [243]. In Table 4, the effects of melatonin supplementation on sperm are summarized against the negative impacts of pollutants.

## 9. Future Perspectives

In the future, research emphasis should be placed on the mechanisms that activate the endogenous production of antioxidants, such as melatonin, to naturally protect sperm from OS [197]. An interesting case is the endogenous production of melatonin in the digestive tract through feeding protocols where varied plans of feeding schedules, different qualities of supplied food, and different sources of natural plant extracts (phytomelatonin) will be tested [244,245,246,247].

Additionally, the use of tryptophan as a dietary supplement could increase melatonin synthesis in the gut and by extension in the blood [245,248]. The important thing, in this case, is that the synthesis of melatonin does not depend on darkness and that the availability and quality of the food provided can affect its synthesis within the gut tissue [249]. On the other hand, the presence of increased levels of phytomelatonin in the blood is accompanied by an increase in serum antioxidant capacity [98,250,251]. More recently, Victoria Peña-Delgado et al. [252] found, for the first time, that a phytomelatonin-rich diet (including a mix of grape pulp, pomegranate, and tomato pomaces) can increase melatonin levels in seminal plasma of ram sperm, improving sperm viability and morphology and protecting sperm cells against oxidative damage. 

Interestingly, from the nutrigenomics point of view, nutrients are feeding signals, which are detected with the cellular system of sensors and influence the expression of genes and proteins, and in consequence, the production of metabolites [253]. Nowadays, nutrigenomics using omic’s technologies investigates molecular relationships between nutrients and genes to identify how even minor modifications could potentially alter animal health and performance [254,255]. However, such types of experiments using gut melatonin and phytomelatonin by emphasizing nutrigenomics, reproduction, and antioxidant capacity in different species is still missing in the available literature.

Another approach, which is quite interesting, is the protective effect of endogenous-produced melatonin on sperm quality. The moment of the highest melatonin concentration in the bloodstream and seminal plasma varies between species, but normally, it rises during the dark period and falls to basal levels during the day [256,257,258]. Félix et al. [257] suggested that endogenously produced melatonin at mid-dark moment of the day may contribute to the improvement of some sperm parameters. In this way, this allows the aquaculture and livestock sectors to select sperm quality sperm by choosing the best moment of the day. 

## 10. Conclusions

There are a variety of situations (heat stress, assisted reproductive technologies, and cryopreservation) that generate RONS in male germ cells. The increase in RONS above a threshold is not physiological and is harmful to the antioxidant defense system of males susceptible to OS, and antioxidants such as melatonin should be a significant factor in preserving the functional integrity of the sperm. Notably, the diet enriched by tryptophan and phytomelatonin supplementation and the suitable time of sperm collection may develop as useful techniques to increase endogenous melatonin in the gut and serum, respectively. Thereby, more carefully controlled research using animals representing different groups of vertebrates is required in the field of the use of endogenous and exogenous melatonin as an antioxidant.

## Figures and Tables

**Table 1:** Effects of melatonin supplementation on sperm quality under heat stress conditions.

Heat Stress Species	Melatonin Concentration	Εffects on Sperm	References
*Human*	1000 μM	Improvement of motility and DNA integrity, reduction in mitochondrial ROS content, lipid peroxidation products and apoptosis, stabilization of mitochondrial membrane potential	[134]
		Improvement of motility, reduction in immobile spermatozoa	[135]
	2 mM	Improvement of motility, elimination of NO–ROS generation	[136]
*Mouse*	-	Reduction in endoplasmic reticulum stress (ERS) signaling pathways and spermatocyte apoptosis	[137]
	-	Testicular recovery	[138]
*Ram*	-	Improvement of progressive and total motility, reduction in abnormalities, increase in acrosome integrity	[139]

**Table 2:** Effects of melatonin supplementation on sperm against the negative impacts of ART stress.

ART Stress	Species	Melatonin Concentration	Εffects on Sperm	References
IVF	*Human*	6 mM	Reduction in apoptosis	[183]
		1 mM	Prevention of DNA fragmentation	[184]

**Table 3:** Effects of melatonin supplementation on sperm against negative impacts of cryopreservation.

Cryopreservation Species	Melatonin Concentration	Εffects on Sperm	References
*Buffalo*	1 mM	Improvement of motility and viability, reduction in apoptotic spermatozoa	[210]
	0.1 mM	Improvement of motility, viability, acrosomal integrity, total antioxidant capacity, superoxide dismutase, and glutathione reductase activity, reduction in lipid peroxidation, aspartate aminotransferase, alanine aminotransferase, alkaline phosphatase, and DNA fragmentation	[211]
*Bovine* *Ovine*	10^−3^ M	Improvement of progressive motility, plasma membrane integrity, mitochondrial membrane integrity, and acrosome integrity	[212]
	0.1 μM, 1 μM, 10 μM, and 100 μM	Increase in motility, decrease in DNA fragmentation	[213]
*Human*	0.1 mM	Upregulation of the expression of heat shock protein (HSP) 90, resistance to stress factors in frozen–thawed sperm	[214]
	3 mM	Improvement of viability and motility, reduction in intracellular ROS level	[215]
*Prochilodus lineatus*	1 mM, 2 mM, and 3 mM	No effects	[235]
	2 mM	Higher curvilinear velocity (VCL) and linear velocity (VSL), reduction in catalysis (CAT)	[216]
*Senegalese sole*	10 mM	Reduction in viability	[217]
*Brycon orbignyanus*	2 mM	Improvement of kinematic parameters	[218]
	2 mM	Better sperm characteristics, peroxidation lipid, and antioxidant enzyme activity	[236]
*Sparus aurata*	0.5 mM, 1 mM, 1.5 mM and 2 mM	No effects	[219]
*Pig*	1.0 μM	Higher viability and acrosome integrity, lower levels of peroxynitrite, ⋅O^2−^ and lipid peroxidation, and total ROS	[220]
*Goat*	0.5 mM, 1 mM, 2 mM, and 4 mM	No effects	[221]

**Table 4:** Effects of melatonin supplementation on sperm against stress of pollutants.

Pollutant Stress Species	Melatonin Concentration	Εffects on Sperm	References
*C57BL/6 J* mice	-	Protection of spermatogonia against the damage and against the histone modification changes induced by hexavalent chromium *Cr* (VI)	[239]
Golden hamster (*Mesocricetus auratus*)	10 mg/kg BW/day	Stimulation of antioxidant proteins Nrf-2/HO-1, SIRT-1/ FOXO- 1, improvement of sperm quality parameters and restoration of changes in testis histomorphology, enhancement of the action of SOD and CAT enzymes, antiapoptotic activity against toxic effects of Bisphenol S (BPS)	[242]
*Wistar* rats	3 mg/L	Weakening of *Cd* toxicity in genes *DAAM1* and *PREP* expression in the testes, reduction in oxidative stress and apoptosis, restoration of histological and biomolecular changes in normal levels, improvement of impaired steroidogenesis (reduced protein level of StAR and 3β HSD)	[243]
